# Lead and Other Trace Elements in Danish Birds of Prey

**DOI:** 10.1007/s00244-019-00646-5

**Published:** 2019-06-18

**Authors:** Niels Kanstrup, Mariann Chriél, Rune Dietz, Jens Søndergaard, Thorsten Johannes Skovbjerg Balsby, Christian Sonne

**Affiliations:** 1 Department of Bioscience, Aarhus University, Grenåvej 12, 8410 Rønde, Denmark; 2 National Veterinary Institute, Technical University of Denmark, Kemitorvet, 2800 Kgs. Lyngby, Denmark; 3 Department of Bioscience, Aarhus University, Frederiksborgvej 399, 4000 Roskilde, Denmark

## Abstract

Lead is a widely used and toxic heavy metal that poses a serious hazard to wildlife species and their ecosystems. Lead is used for production of hunting ammunition. Via gunshot or rifle projectiles, it spreads in ecosystems and may end up in predators and scavengers feeding on wounded or dead animals shot with lead-based ammunition. To assess to what degree Danish raptors are subject to lead contamination, we measured the content of lead in liver tissue from Danish birds of prey (*n* = 137). Additionally, the study included values for 54 other trace elements. In our analysis, emphasis was put on interpretation of lead levels. Levels of cadmium, mercury and selenium were also discussed, while data for the remaining elements were provided for reference purposes. Bismuth was included to assess if lead originated from bismuth gunshot used as an alternative to lead shot. Concentrations of lead, cadmium, mercury and selenium were generally below the levels in similar studies of birds of prey in other northern European countries and none exceeded known and generally accepted threshold values for adverse health effects. As for lead, this is possibly related to the phase out of lead shot for hunting since 1986. The study confirms results from other studies showing that bismuth shot contains traces of lead that is deposited with bismuth in the target animal.

**Electronic supplementary material:**

The online version of this article (10.1007/s00244-019-00646-5) contains supplementary material, which is available to authorized users.

Lead has been used to produce ammunition for military and civilian purposes including hunting for more than 500 years. Lead is a toxic substance, and within the last 40–50 years, there has been an increasing focus on lead poisoning from leaded hunting ammunition (Watson et al. 2009; Delahay and Spray 2015; Thomas et al. 2015; Kanstrup et al. 2019). The main attention to lead exposure in wildlife has been paid to the risk of poisoning of waterbirds ingesting lead pellets as grit and food (Pain 1992). Over the last 10 years, however, there has been an increasing focus on the impact of ammunition lead in other groups of wild birds including birds of prey. It has been documented that predators and scavengers are exposed to lead fragments from rifle ammunition in offal from or carcasses of killed game animals (Kenntner et al. 2001; Helander et al. 2009; Krone et al. 2009; Pain et al. 2010; Nadjafzadeh et al. 2013, Ecke et al. 2017; Pain et al. 2019). Additionally, lead ammunition is a hazard for the health of humans who frequently consume hunted game (Tsuji et al. 2009; Knutsen et al. 2015; Gerofke et al. 2018, Green and Pain 2019). Currently, international scientific literature represents 500–600 studies that support the environmental and health risk of lead in hunting ammunition (Arnemo et al. 2016).

When lead gunshot penetrates a target, traces of microscopic fragments of metal are deposited in meat, connective tissues, organs, blood and bones (Grund et al. 2010; Kollander et al. 2017). Animals with embedded shot may be left in nature (wounded or killed and non-retrieved) and can thus be preyed upon and ingested by predators and scavengers (Arnemo et al. 2016, Pain et al. 2019). Also, birds that ingest gunshot spread through hunting are a source of lead to enter the food chain (Pain et al. 2009). Lead-based rifle projectiles are designed to fragment upon impact with the targeted wildlife specimen to increase the killing efficacy (Kanstrup et al. 2016). The widespread dispersion of metal fragments in carcasses killed with lead-based rifle bullets has been demonstrated in several studies. Hunt et al. (2009) found that all carcasses in a sample of 30 white-tailed deer showed metal fragments. Cornatzer et al. (2009) showed that 59 of 100 randomly selected packages of ground venison were contaminated with lead fragments. In terms of quantity, the risk of environmental lead exposure from an animal shot with a typical lead rifle projectile is high as the offal often is left in the nature. The amount from just a single deer may contain several grams of lead fragments ranging from visible sizes to nanoparticles (Kollander et al. 2017), hence constitutes a potentially toxic dose to several individual scavengers.

The poisoning risk of lead ammunition is well documented internationally (Pain et al. 2019). In Denmark, previous studies of the risk to waterbirds have shown a high prevalence of ingested lead shot and accordingly high mortality in mute swan (*Cygnus olor*) where autopsy of 298 birds showed that 78 (26%) had died from lead poisoning (Clausen and Wolstrup 1979). However, the risk of lead from gunshot and rifle projectiles to expose predators and scavengers through wounded game animals and from offal is poorly investigated in Denmark. The increasing population of deer, the increased opportunities for hunting and the local need for targeted population control combined with the current handling of offal from killed animals accentuate the need to investigate the risk related to leaded rifle ammunition.

The present study was designed to mainly evaluate levels of lead in Danish birds of prey based on liver samples collected as a spin-off of passive toxicological monitoring of wild animals in Denmark. The methodology offered additional measurement of 54 other trace elements, and out of these, we found it feasible to discuss and evaluate cadmium, mercury and selenium, too, because these are commonly regarded to be trace elements to cause adverse impacts in predator and scavenging species. Consult the electronic supplementary material (ESM) for further information on all chemical data.

## Materials and Methods

### Sampling

The material collected for the study included liver samples from a total of 137 Danish birds of prey distributed on 13 species collected by the Technical University of Denmark in the period 2013–2016. One bird was not attributed to species level but derived from a bird of prey. The birds were submitted to the National Veterinary Institute, Technical University of Denmark, for a veterinary examination as part of the general surveillance of wildlife health. The animals were subjected to necropsy and follow-up diagnostic examination including microbiological examination in the accredited laboratory order to establish the cause of death. From all birds of prey, a minimum of 5 g liver sample was stored at – 20 °C in the tissue bank for scientific use. Liver samples are a key indicator of bioaccumulation (Espín et al. 2016) although this differs between elements. For lead, as an example, liver concentrations are indicative of, and can be used to monitor, short-term exposure. Lifetime accumulation is better reflected in bone lead concentrations (García-Fernández et al. 1995, 1997).

For some individuals, sex (*n* = 118), age (*n* = 43) and cause of death or indications of cause of death (*n* = 109) were recorded. The notification “shot” (*n* = 61) included birds killed as a part of bird-strike prevention around airports or cases where this cause of death was established at necropsy. The majority (80%) of the shot birds originated from Midtjyllands Airport (situated in central Jutland nearby the city of Karup) where the shooting is practiced with bismuth gunshot. For “other” birds (complementary to “shot” birds), the cause of death was only reported occasionally, in most cases, as unknown. Hence, this group may include shot birds but with no certainty of this cause of dead.

### Chemical Analyses

The chemical analyses were conducted at the accredited environmental trace element laboratory at Department of Bioscience in Roskilde. A 1.0 g wet weight liver subsample was cut from the main liver sample and digested in Teflon vials with 8 ml of semi-concentrated (i.e. 33%) nitric acid (Merck Suprapure grade) in a Anton Paar Multiwave 3000 microwave oven (according to the Danish Standard DS 259). The main liver samples were not homogenized in order to minimize the risk of grinding shot fragments into the subsample. The digestion programme used 1000–1400 W power for a total of 60 min. After digestion, digestion solutions were diluted with MilliQ water to 60 g and analysed with inductively coupled plasma mass spectrometry (ICP-MS) (Agilent 7900). Detection limits for the elements were determined as three standard deviations on method blank samples. Three certified reference materials (Tort-3, Dolt-5, and Dorm-4 from National Research Council Canada) were included for QA/QC to check digestion efficiency and measurement accuracy. The measured recovery percentage of the reference materials ranged from 87 to 112% for the elements analysed.

The subsequent data analysis involved lead, cadmium, mercury and selenium, which we consider to be the most important elements for assessment of environmental impact of predators and scavengers. In addition, bismuth was included to assess if lead originated from bismuth gunshot (which may contain traces of lead) that has been used as an alternative to lead shot since mid-1990s. We only made comparisons with other studies when these were based on liver samples. When comparing with data of element concentrations expressed per dry weight basis, we used a conversion factor of 3.6 to calculate the corresponding concentrations on a wet weight basis (based on an average of the dry matter percentage in our measurements). In the statistical analyses, if concentrations were below the detection limit, the values were set at half the detection limit for the element concerned.

### Statistical Analysis

We tested if the concentrations differed between species and between shot and other birds using a general or generalized linear model including both factors in the model. For the post hoc test, we used least square mean differences. Data for lead were log transformed to ensure normal distribution, whereas the concentrations of the other elements were fitted assuming a Poisson distribution because transformation could not normalize the residuals for other elements than lead. We omitted a single outlier that had extremely high lead and bismuth concentrations, presumably due to ammunition fragments embedded in the sample. To test the possibility of contamination from lead residues in bismuth gunshot, we compared lead concentrations in shot birds and other birds by using a nonparametric Spearman rank correlation for each category. The analysis required a nonparametric test since the distribution of bismuth could not be transformed to follow normal distribution. Interspecific differences in levels of lead were tested on the four species with *n* > 7 (white-tailed eagle (*Haliaeetus albicilla*), common buzzard (*Buteo buteo*), red kite (*Milvus milvus*) and kestrel (*Falco tinnunculus*)) using a general linear model, whereas a generalized linear model with a Poisson distribution was used for testing differences in cadmium, mercury and selenium levels. The statistical analysis was made in SAS 9.4 (SasInstitute, Cary, NC) using the packages: “proc glm” and “proc genmod”.

## Results

Lead concentrations differed between the four selected species (*F*_3,101_ = 10.4, *p* < 0.0001). Common buzzards had significantly higher lead concentrations than kestrels (see statistical output in “Appendix 1”). Cadmium concentrations also differed between species (*χ*^2^ = 16.9, *p* = 0.0008). The post hoc pairwise comparisons showed significantly higher cadmium and mercury concentrations in common buzzard compared to kestrel (“Appendix 1”). The mercury level differed significantly between species (*χ*^2^ = 23.6, *p* < 0.001). White-tailed eagles showed significantly higher mercury concentrations than in other species (Annex 1). Selenium values did not differ between species (*χ*^2^ = 3.87, *p* = 0.276). However, the highest concentrations across species were found in white-tailed eagles. The molar ratio of selenium/mercury in white-tailed eagles showed that selenium was in surplus (average 15.72, range 0.87–27.59, *n* = 12). Bismuth concentrations differed significantly between species (*χ*^2^ = 21.3, *p* < 0.001). However, the post hoc pairwise comparisons showed no significant differences between species (Annex 1).

The cause of death (shot or other birds) had no significant effect on the concentrations of lead, cadmium and selenium (Pb: *F*_1,101_ = 0.0, *p* = 0.997; Cd: *χ*^2^ = 2,9; *p* = 0.09; Se: *χ*^2^ = 0.34; *p* = 0.558). Shot individuals had significantly lower levels of mercury than other birds (*χ*^2^ = 4.22, *p* = 0.040), whereas birds shot had significantly higher bismuth concentration than other birds (*χ*^2^ = 107.5, *p* < 0.001).

**Fig. 1:**
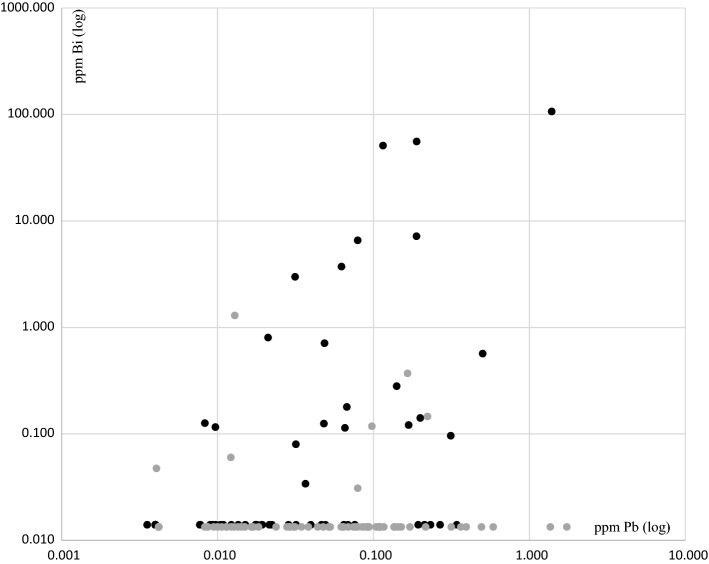
Correlation between lead and bismuth levels for birds reported as shot (black dots) but not for others (grey dots). For shot birds, we found a significant correlation (Spearman correlation, Shot: *r*_s_ = 0.494, *n* = 61, *p* < 0.0001), whereas the relation did not exist for other birds (Spearman correlation *r*_s_ = 0.018, *n* = 75, *p* = 0.877). An extreme value of 15,581 ppm for bismuth in one Kestrel was not included

A positive correlation between lead and bismuth content was observed for shot birds (Shot: *r*_s_ = 0.494, *n* = 61, *p* < 0.0001), whereas this relation did not exist for other birds (*r*_s_ = 0.018, *n* = 75, *p* = 0.877), see Fig. 1.

Table 1 shows the results reported for each species as median, mean and standard deviation (SD) for 136 samples for lead (Pb), cadmium (Cd), mercury (Hg), selenium (Se) and bismuth (Bi). An extreme lead and bismuth value for one kestrel is omitted from the table data. All concentrations are given in ppm (mg/kg) wet weight.

**Table 1:** Median, mean and standard deviation (SD) for five trace elements distributed on species

	N	N_s_	Pb		Cd		Hg		Se		Bi	
			Median	Mean ± SD	Median	Mean ± SD	Median	Mean ± SD	Median	Mean ± SD	Median	Mean ± SD
Common buzzard *Buteo buteo*	48	24	0.09	0.19 ± 0.32	0.38	0.42 ± 0.38	0.32	0.47 ± 0.45	1.20	1.27 ± 0.42	0.01	2.44 ± 15.43
Kestrel *Falco tinnunculus*	39	31	0.02	0.03 ± 0.04	0.03	0.05 ± 0.04	0.12	0.16 ± 0.17	1.05	1.08 ± 0.22	0.01	3.13 ± 11.92
White-tailed sea-eagle *Haliaeetus albicilla*	12	0	0.08	0.24 ± 0.40	0.04	0.05 ± 0.03	1.28	1.92 ± 2.05	2.03	1.93 ± 0.80	0.01	0.01 ± 0.00
Red kite *Milvus milvus*	8	1	0.10	0.11 ± 0.10	0.19	0.15 ± 0.09	0.17	0.16 ± 0.09	1.11	1.07 ± 0.28	0.01	0.02 ± 0.03
Tawny owl *Strix aluco*	6	0	0.02	0.02 ± 0.02	0.43	0.50 ± 0.40	0.16	0.16 ± 0.09	1.30	1.36 ± 0.37	0.01	0.01 ± 0.00
Goshawk *Accipiter gentilis*	5	2	0.01	0.02 ± 0.01	0.05	0.06 ± 0.05	0.25	0.27 ± 0.22	0.89	1.16 ± 0.63	0.01	0.01 ± 0.00
Sparrow hawk *Accipiter nisus*	5	0	0.03	0.04 ± 0.03	0.18	0.18 ± 0.08	1.21	1.66 ± 1.71	0.80	1.07 ± 0.70	0.01	0.01 ± 0.00
Peregrine falcon *Falco peregrinus*	3	0	0.01	0.01 ± 0.01	0.02	0.06 ± 0.08	0.54	0.52 ± 0.35	1.42	1.25 ± 0.37	0.01	0.01 ± 0.00
Eagle owl *Bubo bubo*	3	0	0.01	0.01 ± 0.00	0.07	0.08 ± 0.03	0.51	0.48 ± 0.17	1.00	1.00 ± 0.31	0.01	0.03 ± 0.03
Hen harrier *Circus cyaneus*	2	2	0.00	0.00 ± 0.00	0.07	0.07 ± 0.00	0.17	0.17 ± 0.07	0.74	0.74 ± 0.06	0.01	0.01 ± 0.00
Golden eagle *Aquila chrysaetos*	2	0	0.05	0.05 ± 0.06	0.16	0.16 ± 0.07	0.33	0.33 ± 0.30	0.69	0.69 ± 0.07	0.01	0.01 ± 0.00
Marsh harrier *Circus aeruginosus*	1	1	0.08	0.08	0.03	0.03	0.19	0.19	1.07	1.07	0.01	0.01
Barn owl *Tyto alba*	1	0	0.01	0.01	0.00	0.00	0.03	0.03	0.19	0.19	1.30	1.30
Unknown (bird of prey)	1	0	0.02	0.02	0.36	0.36	3.24	3.24	2.54	2.54	0.01	0.01
	136	61	0.03	0.11 ± 0.24	0.09	0.22 ± 0.30	0.22	0.52 ± 0.91	1.16	1.24 ± 0.51	0.01	1.77 ± 11.12

Unit: ppm (mg/kg wet weight). *N*_s_ = number of birds where the cause of death is reported to be shot. Data for one kestrel with an extreme value of 149 ppm for lead and 15,581 ppm for bismuth were not included

## Discussion

### Lead

Lead from hunting ammunition constitutes a risk for poisoning for a wide range of animal species including predators and scavengers. The relationship is well documented. In 57 white-tailed eagles collected as dead or dying in Germany and Austria, Kenntner et al. (2001) found a mean of 7 ppm lead and a max value of 62 ppm lead and concluded that 28% of the white-tailed eagles had liver lead concentration that could cause acute fatal poisoning (> 15 ppm). The corresponding figure for white-tailed eagles in Poland is 32% (Kitowski et al. 2017) and in Sweden, 12.5% (Helander et al. 2009) and for bald eagles (*Haliaeetus leucocephalus*) in two Great Lakes states, 30% (Nam et al. 2012).

A study of lead in game meat in Denmark found an elevated lead content in pheasants (*Phasianus colchicus*) (Kanstrup 2012). This could be caused by residues of embedded leaded shot; in this case, both lead shot and bismuth shot showed to contain up to 6800 ppm lead. Leaded shot allocates a trace of larger or smaller fragments in the game meat. Bismuth shot is easily fragment causing both bismuth and lead to be released in the target.

A number of studies have established criteria for assessing the risk of lead exposure. Herring et al. (2017) summarized the most recent studies and found for liver samples (wet weight): background: < 2.0 ppm; subclinical: 2.0–6.0 ppm; clinical toxicity: 6.0–15.0 ppm; severe clinical poisoning: > 15.0 ppm. Of all the samples in the present study, only five lead concentrations exceeded 1 ppm and only one exceeded the criterion for subclinical poisoning (2 ppm). This was an extreme value of 149 ppm, which exceeds the limit of acute poisoning of birds by a factor of 10. This value was found in a kestrel shot at Midtjyllands Airport. The bird was an adult female of 202 grams with no reported signs of altered behaviour. Kanstrup (2012) reported values up to 119 ppm lead in pheasants and concluded that lead from embedded bismuth shot could explain the high lead levels measured. Midtjyllands Airport uses bismuth shot for the control of birds causing risk to air safety (Danish Nature Agency, Midtjylland, personal communication) and it is, therefore, likely that the extreme value of 149 ppm lead in the kestrel was caused by a fragment of a shot. This is supported by the fact that the content was far above the acute poisoning limit and the bird also contained an extreme bismuth value. Kestrels are not, particularly, exposed to lead from ammunition as its prey usually includes only small mammals, lizards or large insects.

### Cadmium

Cadmium is a heavy metal that accumulates in organs, in particular, kidney and liver (Haouema et al. 2006). It can pose a serious health risk and cause kidney and bone damage and cancer (Järup and Åkesson 2009).

The threshold level of cadmium poisoning to birds is considered to be about 40 ppm (wet weight, liver) (Sakshaug et al. 2009). All birds in our study had lower values. For common buzzards in Sicily, Licata et al. (2010) found values that correspond to the values of common buzzards in this study. Licata et al. (2010) did not indicate these cadmium levels as critical. For red kites (*Milvus milvus*), Berny et al. (2015) found significantly higher values for cadmium than measured in this study. For white-tailed eagle in Germany and Austria, Kenntner et al. (2001) found cadmium levels in line with our values for both white-tailed eagles and other species, but some high values exceeded these considerably.

Our results showed slightly elevated cadmium levels in some species, e.g. common buzzards. Cadmium concentrations in common buzzards exceeded levels in kestrels more than tenfold, and this difference was statistically significant (Annex 1). Other studies have shown very high cadmium levels in offal of game animals and concentrations tend to be highest in kidney compared to liver and muscular tissues (Lazarus et al. 2014; Durkalec et al. 2015). These studies indicate that offal from shot game animals may be a cadmium source for scavengers like common buzzards which could be the reason to the concentration we found for this species.

### Mercury

Mercury causes loss of senses, blood parameter changes, altered immune response, kidney function and structure, as well as behavioural changes (Boening 2000). It biomagnifies in the food webs. Predators and scavengers, therefore, have a higher risk of mercury poisoning than animals at a lower trophic level (Dietz et al. 1996, Lourenço et al. 2011). The largest concentrations of mercury are found in predatory fish, predators and birds high in the food chain, including notably fish-eating species. Among the species included in our survey, white-tailed eagles had the highest mercury values. A single bird showed a level of 7.5 ppm. Lucia et al. (2010) indicated mercury levels corresponding to the levels of most species in this study, but for two species of waders (Charadriiformes) levels that were above, approximately in line with levels of the white-tailed eagles in our study. Kenntner et al. (2001) found levels (both mean and max values) for white-tailed eagles, which were approximately half of the values in our study and estimated that liver mercury levels below 10 ppm (wet weight) are not critical. The molar ratio of selenium/mercury in white-tailed eagles in our study showed that selenium was in surplus, which in combination with the relatively low mercury levels observed indicated that these birds were not likely to be at risk of mercury intoxication.

### Selenium

Selenium is an important micronutrient but is toxic in high concentrations especially in aquatic ecosystems and associated organisms (Lemly 1993). Selenium is particularly important in mercury detoxification (Ralston and Raymond 2010). Kitowski et al. (2017) examined common buzzards in East Poland and found an average selenium level of 3–4 ppm dry weight which corresponds to the selenium level in common buzzards in this study. We found the highest selenium levels in white-tailed eagles. However, the level is lower than the reported limit of possible harmful effects in waterbirds at 10 ppm (Lemly 1993).

### Lead in Gunshot

All the investigated bird species, except kestrel, may feed on small game subject of hunting with gunshot. Whether the low lead levels is due to the Danish ban on lead shot since 1996 or if the birds of prey are not exposed via small game cannot be concluded. However, studies in other countries show a clear link between use of lead shot for hunting and lead poisoning of birds of prey. Fisher et al. (2006) found exposure to lead in four species of owls and 23 species of birds of prey from North America and Europe. All species listed as exposed to lead from gunshot in Fisher et al. (2006) were included in our study. The low lead levels observed in our study may, therefore, be due to Danish birds not having a source through prey or carrion containing lead gunshot.

Previous studies have demonstrated a significant content of lead in bismuth cartridges up to 0.7% (Kanstrup 2012). We found an extreme lead concentration in one bird along with a very high bismuth concentration. This indicates that the bird was shot with bismuth shot and that the lead in the sample is most likely due to residues of the shot (as opposed to bio-accumulated lead). Preparation of samples by blending or homogenization samples in a mortar in order to achieve a more representative sample will result in a dispersion of metal fragments contained in ammunition residues in the sample. Consequently, in case of birds killed with gunshot, we recommend minimizing this risk of contamination by avoiding homogenization of the samples. This applies, in particular, to tissues that show macroscopic damage caused by gunshot.

Lead in bismuth shot may pose a risk of lead poisoning of birds according to the same pattern as poisoning by pure lead shot. However, Kanstrup (2012) estimated that at a lead content of up to 1%, the release of lead from bismuth shot would pose a negligible risk for birds ingesting such bismuth shot. It has not been evaluated whether exposure from lead and, possibly, other trace elements in shot used as alternatives to typical lead shot represent a health hazard to consumers of game meat or for the environment. Tungsten in gunshot has been suspected to be carcinogenic (Kalinich et al. 2005; Bank-Mikkelsen 2014). However, pure tungsten has not been shown to exhibit carcinogenic properties when ingested or embedded in animal tissues, but nickel, with which it is often alloyed, has known carcinogenicity properties (Thomas et al. 2009). Fäth et al. (2018) showed that the release of certain metals from non-lead shot types dispersed in wetlands was a greater risk for aquatic organisms than lead from lead shot. The main concern here is zinc and copper, whereas, for example, bismuth is not considered to be dangerous in the environment. Even though the lead content in bismuth shot may not constitute a hazard for acute poisoning attention should still be paid to enforce the limits for lead in products as well other hazardous substances in non-lead ammunition.

### Lead in Rifle Bullets

This study did not document any serious contamination of Danish birds of prey caused by leaded rifle ammunition as documented for *w*hite-tailed eagle and golden eagle (*Aquila chrysaetos*) in Germany and Sweden (Kenntner et al. 2001; Krone et al. 2009; Helander et al. 2009). Our survey included 12 white-tailed eagles and two golden eagles, which all showed lead values below background levels. However, our results indicated that lead levels in scavenging species (e.g. common buzzards and red kites) exceeded levels in typical predators (hawks and falcons). For common buzzards, levels were significantly higher e.g. kestrel (Annex 1). This may be explained by exposure from the scavenging species feeding on remains of game animals shot with lead ammunition. In Denmark, leaded rifle ammunition is still legal and widely used for deer hunting. Deer populations presently increase considerably in size and distribution. This is accompanied by increased hunting and population control, hence correspondingly increased use of rifle ammunition. Dispersal of lead from rifle ammunition can be reduced if offal from lead shot animals is removed or left inaccessible for scavengers, i.e. buried. Safe handling of meat and remains from animals shot with lead ammunition, and in particular, phasing out all leaded ammunition will minimize the risk of poisoning of species and contamination of ecosystems in the future.

### Considerations

Apart from a single extreme value for lead, the observed levels of investigated trace elements in this study are below generally accepted threshold levels and thus not considered critical in terms of impact on behaviour, reproduction or survival of the birds. This applies to birds that have been shot for bird-strike control in airports as well as moribund/found dead birds. As lead gunshot is forbidden for hunting in Denmark, and the regulations are largely enforced and complied with (Kanstrup and Balsby 2019), the exposure to ammunition lead is expected to arise only from fragments of leaded rifle bullets in larger game animals. For species that predominately feed on non-hunted species (small birds, rodents, etc.), there is no expected source of ammunition lead. This is complicated by several factors, in particular, that most birds of prey in Denmark are opportunistic and feed depends on season and may consist of both prey and carrions. Furthermore, some of the species included in the study are migratory and may be exposed to lead from ammunition and other sources in other countries at the flyway.

## Conclusion

The examined birds of prey had concentrations of lead, cadmium, mercury, and selenium below the levels demonstrated in comparative studies of birds of prey in other countries and generally below levels considered to be at risk for the bird’s health, behaviour, reproduction and for sustaining a favourable conservation status. As for lead, the low concentration is possibly related to the phase out of lead shot for hunting since 1986. However, the results indicated that lead levels in scavenging species exceeded levels in typical predators which may be explained by continued exposure from the scavenging species feeding on remains of game animals shot with leaded rifle ammunition as demonstrated in other Northern European countries. There is a correlation between lead and bismuth content in birds reported as shot. This confirms results from other studies showing that bismuth shot contains traces of lead that is deposited with bismuth in the target animal. Attention should be paid to enforce the limits for lead in products as well as other hazardous substances in non-lead ammunition.

### Electronic supplementary material

Below is the link to the electronic supplementary material.

Supplementary material 1 (DOCX 18 kb)

Supplementary material 2 (XLSX 84 kb)

## Appendix 1: Statistical Output

See Table 2.

**Table 2:** Post hoc least square means differences and estimates between species for each element

					Least square mean estimate
	WTE	CB	RK	K	
Log Pb					
WTE		0.9362	0.5892	0.0016	− 2.5148
CB			0.4838	< .0001	− 2.4784
RK				0.0205	− 2.8426
K					− 4.1522
Cd					
WTE		0.067	0.429	0.742	− 3.4804
CB			0.18	0.015	− 0.9239
RK				0.53	− 2.1831
K					− 2.9529
Hg					
WTE		0.0013	0.009	0.0003	0.212
CB			0.144	0.062	− 0.8261
RK				0.697	− 2.1549
K					− 1.7624
Bi					
WTE		0.798	0.775	0.803	− 2.0008
CB			0.138	0.702	0.1831
RK				0.142	− 4.6113
K					0.1336
Se					
WTE		0.132	0.127	0.078	0.6328
CB			0.561	0.486	0.2409
RK				0.869	0.02475
K					0.0914

*WTE* white-tailed eagle (*Haliaeetus albicilla*); *CB* common buzzard (*Buteo buteo*); *RK* red kite (*Milvus milvus*); *K* kestrel (*Falco tinnunculus*)
