# Supplementary material for: Lead and Other Trace Elements in Danish Birds of Prey
Source: Arch Environ Contam Toxicol. 2019 Jun 18;77(3):359–67. doi: 10.1007/s00244-019-00646-5 (PMC6731194; doi:10.1007/s00244-019-00646-5)
Supplement: Supplementary file 1 — Supplementary material 1 (DOCX 18 kb) [file 244_2019_646_MOESM1_ESM.docx]

**Electronic supplementary data**

# Article: Lead and other trace elements in Danish birds of prey

Niels Kanstrup^1,*^, Mariann Chriél^2^, Rune Dietz^3^, Jens Søndergaard^3^, Thorsten Johannes Skovbjerg Balsby^1^, Christian Sonne^3^

^1^ Aarhus University, Department of Bioscience, Grenåvej 12, 8410 DK-Rønde. Email: nk@bios.au.dk

^2^ Technical University of Denmark, National Veterinary Institute, Kemitorvet, DK-2800 Kgs. Lyngby, Denmark.

^3^ Aarhus University, Department of Bioscience, Frederiksborgvej 399, DK-4000 Roskilde.

* Corresponding author. E-mail: [nk@bios.au.dk](mailto:nk@bios.au.dk). Tel: +45 20 33 29 99.

**Background**

This dataset represents results of ICP-MS measurements of 55 trace elements in liver samples from 137 Danish birds of prey (13 species) collected by the Technical University of Denmark in the period 2013-2016. The measurements were made as a part of an evaluation of lead from ammunition as a source of lead in birds of prey. The study also included an evaluation of mercury, cadmium, selenium, and bismuth concentrations. As for the other measured trace elements, the study did not make any closer analysis. On this background, we hereby provide the data for other studies.

**Experimental Design, Materials, and Methods**

The chemical analyses were conducted at the accredited environmental trace element laboratory at Department of Bioscience in Roskilde. A 1.0 g wet weight liver subsample was cut from the main liver sample and digested in Teflon vials with 8 ml of semi-concentrated (i.e. 33 %) nitric acid (Merck Suprapure grade) in a Anton Paar Multiwave 3000 microwave oven (according to the Danish Standard DS 259). The main liver samples were not homogenized in order to minimize the risk of grinding shot fragments into the subsample. The digestion program used 1000-1400 W power for a total of 60 minutes. After digestion, digestion solutions were diluted with MilliQ water to 60 g and analyzed with Inductively Coupled Plasma Mass Spectrometry (ICP-MS) (Agilent 7900). Detection limits for the elements were determined as 3 standard deviations on method blank samples. Three certified reference materials (Tort-3, Dolt-5, and Dorm-4 from National Research Council Canada) were included for QA/QC to check digestion efficiency and measurement accuracy. The measured recovery percentage of the reference materials ranged from 87-112% for the elements analyzed.

**Acknowledgements**

We thank Elisabeth Holm, Technical University of Denmark, for liver samples from necropsied birds, For funding we acknowledge The Danish Environmental Protection Agency (grant to the Danish Academy of Hunting, November 2017) and BONUS BALTHEALTH that has received funding from BONUS (Art. 185), funded jointly by the EU, Innovation Fund Denmark (grants 6180-00001B and 6180-00002B), Forschungszentrum Jülich GmbH, German Federal Ministry of Education and Research (grant FKZ 03F0767A), Academy of Finland (grant 311966), Swedish Foundation for Strategic Environmental Research (MISTRA), the Ministry of Environment and Food of Denmark (grant numbers MST-2013/S 080 135070) and by the National Veterinary Institute.

**References**

Kanstrup, N., M. Chriél, R. Dietz, J. Søndergaard, T.J.S. Balsby, and C. Sonne. 2019. Lead and other heavy metals in Danish birds of prey. Submitted June 2019.
